# Caring for the ‘Heads-Down Generation’: Screen Time and Physical Health Complaints Among Adolescents in Poland

**DOI:** 10.3390/jcm15083130

**Published:** 2026-04-20

**Authors:** Joanna Mazur, Alicja Kozakiewicz, Katarzyna Porwit, Dorota Kleszczewska, Maciej Białorudzki, Zbigniew Izdebski

**Affiliations:** 1 Department of Humanization of Medicine and Sexology, Collegium Medicum, University of Zielona Gora, 65-046 Zielona Gora, Poland; a.kozakiewicz@inz.uz.zgora.pl (A.K.); zbigniew.izdebski@uw.edu.pl (Z.I.); 2 Centre of Migration Research, University of Warsaw, 02-093 Warsaw, Poland; katarzyna.porwit@uw.edu.pl; 3 Departament of Health Sociology, Education and Medical Communication, Institute of Mother and Child, 01-211 Warsaw, Poland; dorota.kleszczewska@imid.med.pl; 4 Department of Biomedical Aspects of Development and Sexology, Faculty of Education, University of Warsaw, 00-561 Warsaw, Poland; m.bialorudzki@uw.edu.pl

**Keywords:** screen time, adolescents, physical health complaints, HBSC, social media, eye strain, musculoskeletal pain, sedentary behavior

## Abstract

**Background/Objectives:** Digital media play an important role in the lives of contemporary adolescents. While associated with many benefits, they also pose risks to physical health related to prolonged screen time and non-ergonomic body posture. This study analyzed the frequency of self-reported physical complaints among Polish adolescents in relation to time spent on different screen-based activities. **Methods:** The study included 9083 students aged 13–17 who completed an online survey in March and April 2024 in schools located in western Poland (approximately 30% of the region’s student population). Physical symptoms selected from the HBSC-SCL instrument were analyzed and supplemented with neck or shoulder pain and eye strain. **Results:** Longer screen time was associated with more frequent occurrence of all analyzed complaints. A 5-item index ranging from 0 to 20 points was proposed, including headache, neck or shoulder pain, eye strain, dizziness, and problems falling asleep (mean 6.56 ± 5.15). The index showed reliability at the level of α = 0.744 and good model fit according to CFA (RMSEA = 0.025). In a multivariate linear regression model (*R*^2^ = 0.153), after adjusting for age, gender, place of residence, and family affluence, the variability of this index was most strongly associated with time spent on social media (*β* = 0.40) and browsing websites (*β* = 0.30). Gender-specific models were also compared. **Conclusions:** The results confirm the co-occurrence of physical complaints during adolescence and a significant association between their severity and screen-based activities, particularly engagement in social media.

## 1. Introduction

In large-scale school-based population studies, recurrent non-specific health complaints are commonly used as indicators of physical and psychosocial problems among adolescents [1]. Even in the absence of clinically diagnosed conditions, adolescents frequently report physical symptoms that may reflect physiological responses to stress or overload. These symptoms may include chronic fatigue, sleep disturbances, nausea, dizziness, trembling of the hands, increased heart rate or palpitations, pain in different parts of the body (e.g., headaches, abdominal pain, or back pain), as well as more complex somatic manifestations such as paresthesia, fainting, or seizures [2].

A separate category includes emotional symptoms and cognitive difficulties, such as nervousness, anxiety, low mood, or problems with concentration. However, only a proportion of adolescents undergo detailed diagnostic evaluation or receive medical or psychological care for these complaints; this is likely influenced by the severity and burden of symptoms, as well as adolescents’ willingness to report their difficulties [3]. These manifestations are often described as psychosomatic, reflecting the close interaction between psychological processes and physical health. Psychosomatic problems manifest as physical complaints that originate from psychological processes; however, not all physical symptoms are psychosomatic. When recurrent symptoms raise concerns among adolescents and their parents and interfere with daily functioning, the adolescent is typically first referred to a pediatrician or psychologist and, if necessary, subsequently to other medical specialists (e.g., a cardiologist or neurologist).

The term psychosomatic complaints may be considered outdated in recent literature as there are several other definitions frequently used in recent literature to describe functional symptoms more broadly [4,5]. However, in this paper the authors retain the psychosomatic terminology as direct reference to the HBSC study protocol and the HBSC scale to avoid any methodological misunderstanding [6]. It is worth noting that the population of children with functional disorders is increasing, and this rise is often linked to the overuse of smartphones by adolescents, which emit blue light, as well as rapidly changing content that negatively affects the child’s brain [7]. The onset of serious symptoms, such as seizures, convulsions, or limb weakness, often require urgent medical attention [8]. This may lead to the need for a long-term treatment [9]. A better understanding of the behavioural risk factors associated with screen time related disorders, combined with appropriate educational efforts, may help modify behavioural patterns before clinical intervention becomes necessary.

Various instruments are used in population studies to measure the frequency of recurrent health complaints, as well as their determinants and consequences. These instruments typically consist of symptom lists that are defined and categorized according to different criteria. Examples include the Children’s Somatic Symptoms Inventory (CSSI-24) [10], the somatic complaints subscale of the Child Behavior Checklist (CBCL) [11], the Brief Symptom Inventory (BSI) [12], and the Health Behavior in School-aged Children Symptom Checklist (HBSC-SCL) [13]. The literature is particularly rich in studies using the latter scale due to the wide coverage and repeated cross-sectional design of the Health Behavior in School-aged Children (HBSC) study. Among other applications, the HBSC-SCL scale has been used to analyze social inequalities in the health of school-aged adolescents [14,15], to examine the relationship between health complaints and leisure-time activities [16,17], physical activity [18], dietary habits [19], and risk behaviors [20,21].

Based on the results of HBSC studies involving students from 44 countries, the criterion for the occurrence of recurrent psychosomatic complaints was met by 66% of girls and 36% of boys aged 15 who were surveyed in the 2021/22 school year [22]. In the same study, Polish adolescents ranked between third and fifth place, depending on the age group, in terms of decreasing rates of multiple health complaints. Subsequent international HBSC reports confirm an increase in the frequency of reported multiple complaints with age and a higher burden among girls than among boys [22,23]. In most countries, successive waves of HBSC surveys have shown an upward trend in reported complaints. These increasing trends have been attributed to the global adolescent mental health crisis [24], changes in lifestyle [25], including increased engagement with social media [26], and more recently to experiences associated with the COVID-19 pandemic [27].

The present study uses data from a survey based on the HBSC methodology (procedure, age groups, research instruments), conducted in one region of Poland outside the main schedule of the international surveys. The analysis examines how screen time relates to selected health complaints. Although negative health consequences of prolonged screen time may occur across different age groups [28], children and adolescents require particular attention. They represent the first generation to grow up in an environment shaped by modern information technologies, which creates opportunities for development but also introduces new health risks [29]. Psychosocial risks arise from several mechanisms. First, the transfer of social relationships to the internet may paradoxically intensify loneliness. Second, reliance on online resources may limit developmental experiences. Third, adolescents may be exposed to harmful online content, such as hate speech, cyberbullying, or sexual harassment. Another concern involves the risk of developing addictions (e.g., to computer games, social media, or smartphones), violations of personal data privacy, exposure to marketing activities, and the related risk of online fraud. Threats to physical health, in turn, are associated with the lack of ergonomic practices when using computers or smartphones, poor sleep and rest hygiene, and the reduction or abandonment of physical activity [30].

The term heads-down generation is increasingly used in the literature to describe individuals who spend a significant proportion of their time using mobile devices with their heads bent forward [31]. Individuals using smartphones often adopt a characteristic posture and gait involving a forward head tilt, a relatively rigid neck, rounded shoulders, and arms held close to the torso. At the same time, it is emphasized that the widespread availability of these devices may encourage situations in which individuals focus on virtual interactions at the expense of their immediate social environment [32]. This phenomenon is also analyzed in the context of so-called text/tech neck and other physical complaints associated with prolonged exposure to screen time [33]. Prolonged forward head posture may also trigger compensatory changes in the thoracic spine, including increased kyphosis (slouching), which is often accompanied by a forward shoulder position and may contribute to scapular instability due to weakening of the serratus anterior muscles. These tendencies reflect broader changes in patterns of Internet use that have intensified with the development of mobile technologies. According to the Polish EUKids Online study conducted in 2018, adolescents primarily access the Internet via smartphones, which limits parents’ ability to monitor both the time spent using the device and the types of activities undertaken online [34]. Increasing attention is also paid to the fact that interaction with the digital environment shapes the human brain and alters the rules of social communication [35]. Consequently, initiatives have emerged to introduce legal regulations restricting Internet access and banning smartphone use in schools, as well as technological solutions that set time limits or block certain applications and websites. Guidelines are also being developed on the safe use of computers and mobile devices and on fostering digital awareness from an early age [28]. In addition, many countries promote age-appropriate recommendations regarding the amount of time that should be devoted to moderate and vigorous physical activity, light physical activity, sedentary leisure activities, and sleep in order to maintain a balance between online and offline life [18,36].

The present study combines an analysis of screen time with the occurrence of exclusively physical complaints and, in line with previous HBSC research, considers a potential dose–response effect [17]. As in other studies, associations with individual complaints [37] as well as with their co-occurrence as a symptom cluster were examined [38]. When designing the questionnaire, it was decided to expand the list of analyzed complaints. The frequency of neck or shoulder pain and eye strain in the studied population was therefore assessed [39]. The relevance of including these symptoms has been confirmed in recent literature reviews [40,41,42,43]. We propose an alternative measure to the physical subscale of the HBSC-SCL that may be useful in analyses of health consequences associated with prolonged screen time and problematic smartphone use.

The aim of the study was to analyze the association between screen time and complaints reported by adolescents and to construct a new cluster of physical complaints that has not been applied in previous analyses. Attention was also paid to the potential harmfulness of different types of screen-based activities and to the possible dose–response effect. Screen time and the burden of complaints were analyzed in relation to selected socio-demographic characteristics, with particular attention to gender differences.

## 2. Materials and Methods

### 2.1. Participants and Procedure

The survey was conducted online in spring 2024 using the Webankieta platform (Warsaw, Poland). Data were obtained from 9411 students from three school cohorts, hereafter referred to as K7, K9, and K11: Grade 7 of primary school (mean age 13.76 ± 0.38 years), the first grade of secondary school (15.20 ± 0.57 years), and the third grade of secondary school (17.67 ± 0.53 years). The sample included approximately 30% of students enrolled in schools in the Lubuskie Voivodeship, a region in western Poland.

The analyses included data from 9083 students who completed all items related to the analyzed complaints and leisure screen time. This group consisted of 45.6% boys and 54.4% girls, and respectively 41.0%, 37.0%, and 22.0% of students from the three cohorts mentioned above. The overrepresentation of girls was more pronounced in the two older cohorts (51.4%, 56.4%, and 57.4% girls in the three age groups, respectively). In the analyzed sample, 21.5% of students lived in large cities, 42.5% in smaller towns, and 36.0% in rural areas. In addition, 34.2% of students came from less affluent families, 49.5% from families with an average socioeconomic status, and 16.3% from more affluent families (according to the criteria described below).

The study procedure and the scope of the collected information were reviewed by a local research ethics committee. Parents of underage students provided consent for their child’s participation through the school administration. Students expressed informed consent by completing the questionnaire and could withdraw at any time. The organization of the study and the data-cleaning process are described in more detail in the national report [44].

### 2.2. Tools and Variables

Six symptoms reflecting bodily or physical complaints were included as dependent variables, although their psychological origin cannot be excluded. Four of these symptoms were derived from the HBSC Symptom Checklist (HBSC-SCL) developed by the HBSC research network [13]. In its original form, the scale includes eight symptoms that can be analyzed either as a one-dimensional construct or as a two-dimensional construct distinguishing psychological and physical dimensions.

The symptoms selected for further analyses from this source were: headache, backache, dizziness, and difficulties in getting to sleep. According to the classification currently used in HBSC studies, the first three are categorized as physical complaints, while the last is considered psychological. However, sleep problems may also be strongly associated with physical symptoms [45].

Two additional symptoms beyond the HBSC-SCL were included in the questionnaire: neck or shoulder pain and eye strain. Neck pain was present in the Norwegian prototype of the HBSC-SCL and has periodically been included in the HBSC protocol as an optional question [13]. Eye strain was introduced as a new symptom and defined as including blurred vision, tearing, and red eyes, in order to distinguish it from visual impairments such as refractive errors or strabismus.

Students assessed the frequency of each symptom during the previous six months using five response categories coded from 0 to 4: rarely or never, about every month, about every week, more than once a week, and about every day. Experiencing a symptom more than once per week was considered frequent.

The main independent variables concerned time spent in front of screens. A block of questions derived from the HBSC 2022 study protocol was used. Four types of leisure-time screen-based activities were distinguished and are referred to below using the abbreviated terms provided in parentheses. These were:

(1) Playing games on a computer, game console, tablet, smartphone, or smart TV (computer games);
(2) Using a computer and other electronic devices for social networks for example Instagram, Facebook, Twitter, Snapchat, etc. (social media);
(3) Watching TV, DVDs, or videos including internet videos on websites like YouTube, etc. (TV or videos);
(4) Looking up information on the internet, browsing the internet (websites).

Screen time was assessed using nine response categories: none at all, about half an hour a day, about 1 h a day, about 2 h a day, about 3 h a day, about 4 h a day, about 5 h a day, about 6 h a day, about 7 or more hours a day. For further analyses, five aggregated frequency categories were distinguished and the time devoted to each activity was estimated in hours. It should be noted that these activities may occur simultaneously; therefore, the reported times do not necessarily sum to the total duration.

The remaining independent variables were as follows: gender (boys, girls), grade (K7, K9, K11), place of residence (cities with more than 100,000 inhabitants, smaller towns, rural areas), and family affluence measured using the Family Affluence Scale (FAS). This scale was developed by members of the HBSC research network and, in its current version (FAS III), consists of six questions concerning household car, computer, dishwasher, and bathroom ownership, having an unshared bedroom, and family holidays abroad [46]. The FAS III score ranges from 0 to 13 points and is divided into three categories corresponding to low, medium, and high family affluence, with cut-off points at 6/7 and 9/10. In the analyzed sample, there were 124 missing values for this variable (1.4%).

### 2.3. Methods of Analysis

The overall distribution of responses to key questions concerning screen time and physical complaints was presented using weighted data, assuming equal numbers in six groups defined by gender and grade (theoretically 1520 individuals in each group). In the univariate analysis, associations between categorical variables were examined using the chi-square test, and Cramer’s V coefficient was reported as a measure of effect size. Differences between means were tested using non-parametric methods for independent samples, namely the Mann–Whitney U test and the Kruskal–Wallis test, depending on the number of groups compared. Post hoc Kruskal–Wallis analyses were conducted for multiple comparisons, with significance levels adjusted using the Bonferroni correction. The association between the frequency of reporting different symptoms was assessed using Spearman’s rank correlation coefficient.

The structure of alternative indices of physical complaints was examined using confirmatory factor analysis (CFA). The quality of the alternative models was evaluated using the following fit indices: χ^2^, CMIN/DF, root mean square residual (RMR), comparative fit index (CFI), normed fit index (NFI), relative fit index (RFI), Tucker–Lewis index (TLI), and root mean square error of approximation (RMSEA) with a 90% confidence interval (CI). Decisions to remove particular items were based on theoretical considerations and modification indices (MI). The reliability of the indices was assessed using Cronbach’s alpha. A summary index of physical complaints was calculated based on the optimal set of items.

In the multivariate analysis, the association between screen time and physical complaints was examined using general linear models (GLM). Gender, age, family affluence, and place of residence were included as fixed factors, while the estimated number of hours spent on specific screen-based activities was included as covariates. Separate linear models were estimated for each symptom and for the optimal summary index. The results of the GLMs are presented as *β* coefficients with 95% confidence intervals and standard errors, their significance levels, and partial eta squared as a measure of effect size. Model fit was evaluated using the *R*^2^ coefficient.

Data were analyzed using IBM SPSS Statistics (Version 29) and IBM SPSS Amos (Version 29).

## 3. Results

### 3.1. Screen Time

Table 1 presents data on the time adolescents spent in front of screens during their leisure time, including activities performed on computers, tablets, or smartphones. The values shown are weighted percentages, assuming equal group sizes by age and gender. The adolescents surveyed reported that they spent the most time on social media and the least time browsing websites in search of information.

An additional summary presented in Appendix A (Table A1) shows the association between screen time and the respondents’ socio-demographic characteristics. Gender differences were observed for three of the four analyzed behaviors. Boys spend more time playing computer games and watching TV or videos, whilst girls devote about 1.5 h more to social media activity. Screen time was also found to be associated with the age of the respondents. The largest difference was observed for computer games, which occupied less time among students in the third grade of secondary school. In the post hoc analysis, the difference between cohorts K7 and K9 was not significant (*p* = 0.098). Time spent browsing websites increased systematically with age, and the post hoc analyses showed statistically significant differences between all cohort pairs (*p* < 0.001). Students from the youngest cohort (K7) spend less time on social media, whereas in the post hoc analysis the difference between K9 and K11 was not significant (*p* = 0.831). For time spent watching TV and video materials, a weak non-linear association with age was observed, with the lowest value recorded in the middle cohort. In the post hoc analysis, the difference between K7 and K9 was closest to the level of statistical significance (*p* = 0.077).

The association between family affluence and time spent on screen-based activities was observed mainly for computer games and watching video materials. In both cases, adolescents from the least affluent families spent more of their leisure time on these activities. In the post hoc analysis, the difference between adolescents from families with average and high affluence was not significant for computer games (*p* = 0.369), whereas for watching videos significant differences were observed between all pairs of affluence groups. Students from the most affluent families tended to be more active on social media; however, the differences between socioeconomic groups were close to the threshold of statistical significance (*p* = 0.056). In the post hoc analysis, the largest difference was observed between cohorts K7 and K11 (*p* = 0.064). The association between screen time and place of residence was most pronounced for the frequency of browsing websites. Adolescents living in small towns searched for information online more often than their peers from large cities and rural areas. The popularity of computer games tended to increase with higher levels of urbanization; however, the observed differences were close to the threshold of statistical significance (*p* = 0.042), with the largest difference in the post hoc analysis being observed between small towns and rural areas (*p* = 0.002).

### 3.2. Frequency of Reported Physical Complaints

Table 2 presents the frequency of individual complaints reported by students during the previous six months, based on weighted data. Difficulties falling asleep and back pain were reported most frequently, whereas dizziness was reported least often.

The frequency of experiencing individual complaints was correlated moderately (given the sample size, *p* < 0.001), with Spearman’s correlation coefficients ranging from 0.285 (eye strain and neck or shoulder pain) to 0.584 (back pain and neck or shoulder pain). It is also noteworthy that dizziness showed relatively stronger correlations with symptoms such as headache (0.532) and difficulties falling asleep (0.429).

Table 3 compares the prevalence of physical complaints across groups defined by socio-demographic characteristics. The comparison focuses on the proportion of adolescents who frequently reported individual symptoms. Girls reported all six physical complaints more often than boys, with the largest gender differences observed for dizziness and headache. The association with age was strongest for back pain and neck or shoulder pain, for which the proportion of adolescents frequently reporting these symptoms increased systematically across successive cohorts. Substantial age-related differences were also observed for dizziness; however, in this case the highest prevalence was found among 15-year-olds (K9). Recurrent eye problems were reported less frequently by students in primary school (K7) than by those in the two older cohorts. No age-related differences were confirmed for difficulties falling asleep or headache, although in the latter case the result was close to statistical significance (*p* = 0.052), with the greatest increase in prevalence observed between the ages of 13 and 15.

The association between recurrent physical complaints and family affluence proved to be relatively weak. Statistically significant differences were confirmed only for dizziness. This symptom was reported least frequently by adolescents from families with an average level of affluence. A similar pattern was observed for three other symptoms (headache, eye problems, and neck or shoulder pain), where the results were close to the threshold of statistical significance (*p* values between 0.063 and 0.081).

Place of residence showed the weakest association with the occurrence of recurrent physical complaints among the analyzed socio-demographic factors. Statistically significant differences were confirmed only for neck or shoulder pain, with the prevalence of this problem increasing with the level of urbanization.

### 3.3. Index of Physical Complaints

To summarize the above analyses, a summary index measuring the intensity of physical complaints among school-aged adolescents was proposed. In the first step, the psychometric properties of an index combining six symptoms were examined using confirmatory factor analysis (CFA). If the results proved unsatisfactory, alternative shorter indices were compared. The results of these analyses are presented in Appendix A (Table A2). All tested solutions demonstrated satisfactory reliability according to Cronbach’s alpha.

The scale containing six symptoms (Index 1) showed unsatisfactory fit indices. It was assumed that this was due to the strong correlation between back pain and neck or shoulder pain. Therefore, two scales including five symptoms were tested, successively excluding neck or shoulder pain (Index 2) and back pain (Index 3). It was found that Index 3 had slightly better model fit parameters. One limitation was the still relatively high CMIN/df value. An improvement in this indicator can be achieved by correlating the error terms for the variables referring to headache and sleeping difficulties (Index 3a).

**Figure 1:**
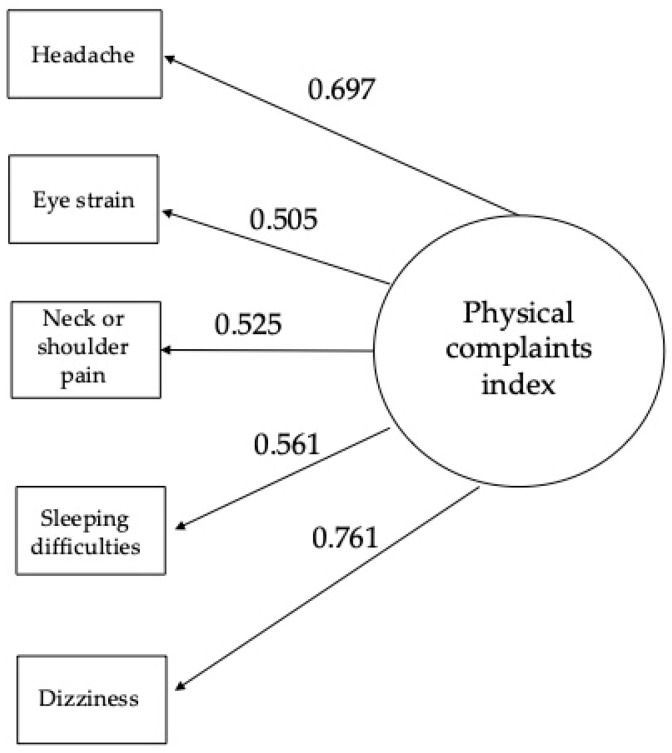
CFA model of the short physical complaints index associated with screen time.

As Index 4, a scale excluding sleeping difficulties—the symptom least directly associated with physical complaints—was also defined. However, this solution did not improve the psychometric properties and resulted in lower reliability. Therefore, subsequent analyses were based on the five-item summary index excluding back pain (Index 3). Figure 1 presents the corresponding CFA model. The standardized regression weights range from 0.505 (eye strain) to 0.761 (dizziness), and are also relatively high for headache (0.697).

The simple summary index ranges from 0 to 20 points. Higher values indicate a greater severity of physical complaints. In the studied sample, the mean value of the index was 6.54 (SD = 5.15). Table 4 presents the distribution of this index according to the socio-demographic characteristics of the surveyed adolescents. Mean values were compared using non-parametric statistical tests. When three groups were compared, post hoc Kruskal–Wallis analyses were conducted.

Significant differences were observed by gender, with girls reporting a higher burden of physical complaints. The severity of physical complaints also increased with age; however, the post hoc analysis did not confirm a significant difference between cohorts K9 and K11. The relationship between the index score and family affluence followed a non-linear pattern. The most favorable results were observed among adolescents from families with average affluence, compared with the two extreme groups, which showed similar levels of the physical complaints index. An association between the level of the examined index and place of residence was also found, favoring students living in rural areas. In the post hoc analysis, no significant difference was confirmed between residents of small and large cities, although the mean values were slightly higher in larger cities.

### 3.4. Association Between Individual Physical Complaints and Screen Time

Inference regarding the relationship between specific screen-based activities and the severity of physical complaints was based on multivariate analysis using GLMs. Table 5 presents the results of model estimations conducted for individual symptoms. Associations with screen time are represented by *β* coefficients, while the strength of these associations is indicated by the partial *η*^2^ index. It was found that, after adjusting for other socio-demographic factors (gender, school cohort, place of residence, and family affluence):

- The severity of all symptoms was most strongly associated with time spent on social media, with the strongest associations observed for dizziness and back pain;
- The weakest associations were observed between the severity of the analyzed symptoms and time spent watching TV or videos; for several symptoms this relationship was not statistically significant (headache, dizziness) or was borderline significant (sleeping difficulties);
- The goodness-of-fit of the individual GLMs adjusted for four socio-demographic factors ranged from 5.1% (neck or shoulder pain) to 11.8% (dizziness);
- The goodness-of-fit of the GLMs including only the four screen-time variables as covariates ranged from 3.2% (neck or shoulder pain and eye strain) to 6.3% (dizziness).

Table 6 presents the results of the GLM model estimation in which the dependent variable is the summary index of physical complaints previously identified as optimal (headache, eye strain, neck or shoulder pain, sleeping difficulties, dizziness). After adjusting the analyses for demographic and social factors, it was confirmed that spending more hours on each of the four types of screen-based activities is significantly associated with an increase in the average score of the physical symptoms index. The strongest association was observed for engagement in social media. A lower burden of this cluster of complaints was also observed among boys compared with girls, and among 13-year-old students compared with 17-year-olds. As in the univariate analysis, the difference between groups K9 and K11 remained non-significant.

In the multivariate model, living in a small town was associated with a higher burden of the physical complaints cluster, whereas the difference between residents of large cities and rural areas was no longer significant (*p* = 0.068). The association between family affluence and the examined cluster of complaints was also less pronounced. In the multivariate model, no significant difference was confirmed between adolescents from families with average and high affluence, although in simple distribution comparisons this difference was significant to the disadvantage of the more affluent group. The model presented in Table 6 explained 15.3% of the variance in the physical complaints index.

Given the strong association between gender and the severity of physical complaints, analogous multivariate models were estimated separately for boys and girls (Table 7). The goodness of fit of both models was similar. After stratifying the analyses by gender, the interpretation of the strength of the association between the severity of physical complaints and screen time changed. For both genders, the strongest association was confirmed for engagement in social media; however, the value of the β coefficient was higher among girls. Only among boys was there no association found with time spent watching video materials, while the association with time spent browsing websites was stronger for boys than for girls. Among boys, age-related differences were more pronounced, favoring 13-year-olds. Among girls, no difference was observed between the two older cohorts (K9 and K11). The difference between residents of small towns and rural areas was significant only among girls, in favor of rural residents. For both genders, family affluence had no significant effect on the variability of the examined index.

## 4. Discussion

In this section, the results of this study are interpreted in the context of current evidence on patterns of digital media use and their potential health consequences. This issue is particularly important given the increasing prevalence of problematic digital media use among adolescents [47]. We analyzed different forms of screen time and their association with physical complaints during adolescence, which aligns with current research examining the links between media use and adolescent health across multiple dimensions [48]. Particular attention was also given to the role of socio-demographic factors, especially gender, as a potential moderator of the observed associations.

### 4.1. Screen-Based Activity Among Adolescents

The results indicate that social media was the dominant form of leisure screen activity among the surveyed adolescents, whereas the least time was devoted to browsing websites for information. The average daily time spent on social media (2.89 h) exceeded that devoted to computer games (2.61 h), watching TV/videos (1.84 h), and browsing websites (1.04 h). The distribution of responses also indicates considerable behavioral heterogeneity, with a subgroup of students reporting very high engagement in social media and computer games (≥6 h per day).

This profile of screen use reflects recent changes in how adolescents engage with digital technologies, characterized by a shift from passive content consumption toward socially interactive and continuously stimulating forms of use [49]. The scale of this global phenomenon is also reflected in data published by Eurostat, indicating that in 2022 more than 80% of adolescents in Europe used the Internet daily to participate in social networking sites [50]. The predominance of social media use over video viewing, combined with the relatively low share of information-oriented activities, suggests that the Internet currently functions mainly as a relational and entertainment-oriented environment [51,52]. It is also important to note that different forms of screen time should not be treated as a homogeneous behavioral construct, as they may be associated with different health outcomes, which justifies analyzing them separately rather than using a single aggregate measure of screen time [53].

The variation in screen time by gender and age indicates that the use of digital technologies during adolescence is shaped by developmental and social factors. Greater engagement of boys in gaming and watching video content, alongside longer time spent by girls on social media, is consistent with findings from other studies, which additionally point to a stronger association between digital media use and reduced psychological well-being among girls [54,55].

Moreover, with increasing age, a shift in adolescents’ screen-based activity patterns can be observed, characterized by a decline in time devoted to computer games and a rise in engagement with social media and browsing websites, as also reported in previous studies [56]. This pattern suggests a transition from primarily entertainment-oriented activities toward more relational forms of digital engagement. At the same time, time spent watching television and video materials appears to remain relatively stable across different stages of adolescence.

In the present study, the association with family affluence was observed mainly for computer games and watching video materials, which more frequently engaged adolescents from families with lower socioeconomic status. This finding is consistent with reports linking lower socioeconomic status to a higher prevalence of sedentary leisure behaviors [57,58]. These results further support the view that screen time should be treated as a qualitatively heterogeneous construct, with patterns that vary according to socio-demographic characteristics.

### 4.2. Physical Complaints

The distribution of reported physical complaints in the present study indicates that a considerable proportion of adolescents’ experience symptoms that are recurrent rather than incidental. The most frequently reported complaints were difficulties falling asleep and back pain, while dizziness was reported least often. The analyses also included less frequently examined symptoms such as eye strain, which may result from both screen light exposure and prolonged visual effort during digital device use.

Moderate but significant correlations between complaints indicate that these symptoms form a cluster of non-specific physical complaints. The particularly strong association between back pain and neck or shoulder pain suggests shared underlying mechanisms, such as prolonged sitting, musculoskeletal strain, and lack of ergonomic conditions in everyday functioning [59]. Moreover, one potential factor contributing to the co-occurrence of these complaints may be the use of mobile devices, especially smartphones, which is associated with prolonged head flexion and strain on the cervical spine (“text/tech neck syndrome”) [60]. In contrast, the correlations between dizziness, headache, and difficulties falling asleep may reflect more complex interactions between somatic symptoms and sleep regulation [61].

From a practical perspective, these findings highlight the importance of preventive strategies aimed at reducing the risk of text/tech neck syndrome. Such strategies include maintaining a neutral head and neck position, positioning the screen at eye level, limiting prolonged static postures, and incorporating regular breaks during screen use. Promoting physical activity and postural awareness among adolescents may further help counteract the musculoskeletal strain associated with excessive use of mobile devices [62].

The results confirm clear differences in the frequency of recurrent physical complaints by gender, age, and, to a lesser extent, socio-economic context. The strongest and most consistent differences were observed by gender: girls reported all analyzed complaints more frequently than boys, with the largest disparities observed for dizziness and headache. This pattern is consistent with numerous studies showing that girls during adolescence experience a higher burden of non-specific physical complaints [6,63].

Age-related associations were more selective and mainly concerned musculoskeletal complaints. The systematic increase in the frequency of back pain and neck or shoulder pain across successive cohorts may reflect increasing postural strain, prolonged sitting, and cumulative exposure to ergonomic risk factors with age [64]. A different pattern was observed for dizziness, which was reported most frequently by adolescents aged 15 years. The absence of clear age-related differences for difficulties falling asleep and headache suggests that these symptoms may have a more stable pattern during adolescence, independent of school age.

Associations between physical complaints and family affluence or place of residence were weaker and less consistent, being limited to a small number of symptoms, such as dizziness and neck or shoulder pain. These findings suggest that during adolescence, gender and developmental stage may play a more important role in the burden of non-specific physical complaints than indicators of socioeconomic status.

### 4.3. Forms of Screen Time and Physical Complaints Among Adolescents

In this study, we analyzed the association between screen time and both individual physical complaints and their cumulative indicator. We applied a summary index of physical complaints, which allowed for an overall assessment of the burden of somatic symptoms among adolescents. The final five-item index derived from factor analysis is justified both statistically and clinically, indicating that the analyzed symptoms more often co-occur as a cluster rather than as independent manifestations [65]. The results indicate a significant cumulative burden of physical complaints, which is higher among girls and increases with age, with a tendency to stabilize in later adolescence.

The exclusion of back pain from the applied summary index may be considered a limitation; however, this does not disprove its connection with screen time. We have demonstrated that back pain and the added symptom of pain in the shoulders and neck are strongly correlated. The second symptom is more strongly associated with the body posture adopted whilst using smartphones. The use of a 6-item scale may be clinically justified, but it has substantially poorer psychometric properties, including RMSEA and SRMR values that exceed the acceptable thresholds.

A particularly important finding concerns the differentiation of associations between physical complaints and specific forms of screen time. The general linear model analysis showed that the strongest associations were related to social media activity. Time spent on social media was significantly associated with all analyzed symptoms, with the strongest effects observed for dizziness, back pain, and difficulties falling asleep. This pattern suggests that interactive activities, which are cognitively and emotionally engaging and often performed in a static body position, may contribute to the accumulation of non-specific physical complaints.

A positive association between social media use and somatic complaints during adolescence has also been demonstrated by Koç et al. [66], Hanprathet et al. [67], and Marino et al. [68], who documented the negative impact of problematic social media use on overall health, including somatic complaints, anxiety, insomnia, depression, and social dysfunction. Similar findings have been reported in Polish studies, which showed that Internet and social media use was most strongly associated with the occurrence of health complaints among adolescents, encompassing all analyzed symptoms [69]. Furthermore, it has been suggested that problematic Internet use may increase the risk of somatization both directly and indirectly through sleep disturbances [70], which may negatively affect self-rated physical health and school functioning [71]. Users exhibiting problematic patterns of social media use also more frequently report mental health complaints, supporting the hypothesis that problematic social media use (PSMU) may contribute to increased symptoms of anxiety, depression, loneliness, and mood instability, particularly among children and adolescents [72,73]. At the same time, it is important to acknowledge that social media use is not exclusively associated with negative outcomes. When used in a balanced and informed manner, it may support social connectedness, access to information, and peer support [74]. Therefore, promoting digital literacy and responsible use of online platforms should be considered an important component of adolescent health promotion strategies [49]. 

Time devoted to computer games was also significantly associated with the severity of most physical complaints; however, the strength of these associations was clearly weaker than in the case of social media use. The most pronounced relationships were observed for difficulties falling asleep, back pain, and neck or shoulder pain, which may reflect both prolonged musculoskeletal strain and cognitive arousal that makes it more difficult for the body to relax [75]. Research also indicates that exposure to blue light emitted by electronic devices may worsen sleep quality by disrupting the physiological mechanisms regulating sleep and rest [76]. Moreover, adolescents who exhibit problematic gaming behaviors have been shown to report poorer health and well-being compared with non-gamers and are more likely to experience sleep disturbances [77]. More recent evidence suggests that psychological distress, including depressive symptoms and negative affect, may increase the risk of developing problematic gaming patterns [78,79].

Time spent browsing websites showed moderate but significant associations with most of the analyzed complaints (with the exception of back pain, which remained non-significant). This pattern may indicate that even activities perceived as relatively neutral or instrumental are associated with prolonged sitting, visual strain, and disruption of circadian rhythms, all of which may contribute to the occurrence of non-specific physical symptoms [53]. 

The least consistent associations were observed for watching television or video materials. For some complaints, including headache and dizziness, no significant relationships were found, and for difficulties falling asleep the result was borderline significant. The weaker links between this form of screen time and physical complaints may stem from its more passive character and lower emotional and interactive engagement compared with other types of digital activities.

Furthermore, the multivariate analysis confirmed that screen time is significantly associated with the severity of the cluster of physical complaints among adolescents. All four forms of screen-based activity contributed independently to explaining the variability of the index, although their relative importance differed considerably. The strongest association was observed for social media activity, which accounted for the largest proportion of variance explained in the index, as discussed above.

### 4.4. Gender Differences

In the GLM model, significant differences related to gender remained evident. The lower index values observed among boys are consistent with previous findings indicating a greater burden of physical complaints among girls. Further analyses showed that in both gender groups the strongest association concerned engagement in social media, although this relationship was clearly stronger among girls. In addition, only among girls was a significant association confirmed between watching video materials and the severity of complaints, whereas among boys this relationship did not reach statistical significance.

Other studies have also shown that social media use is associated with increased internalizing symptoms especially among girl [54], as well as with lower levels of well-being, expressed for example through more severe depressive [80] and anxiety symptoms [81], although not all studies confirm the existence of gender differences [82]. The stronger association between digital media use and poorer well-being among girls [83] may result from different patterns of social media use depending on gender [84,85], as well as from the greater importance girls attach to social relationships [86] and body image [87,88]. Moreover, girls more often publish different types of selfies, use filters, modify photos, and delete their posts more frequently, whereas boys more often update their profiles with content related to sports and technology [80,89].

An opposite pattern was observed for browsing websites, which was more strongly associated with the physical complaints index among boys than among girls. Age-related differences were also more pronounced among boys, with a lower burden of complaints in the youngest cohort, whereas among girls no differences were found between the two older age groups. The effect of place of residence was significant only among girls, favoring those living in rural areas. For both genders, family affluence did not play a significant role.

### 4.5. Strengths and Limitations

A major strength of this study is its large sample size (9411 students). The analyses also incorporated demographic and social factors, with particular attention to gender differences. Compared with some studies examining the determinants of health complaints among school-aged adolescents, however, the number of analyzed factors remains limited. This was partly due to the thematic scope of the questionnaire and partly to the overall design of the planned analyses. An important variable not included in the questionnaire is the presence of chronic diseases, which may be associated with more frequent reporting of the analyzed symptoms [90].

Future analyses using the same 2024 dataset may examine health complaints in relation not only to screen time but also to other factors, such as physical activity, sleep duration, and body mass index. An additional strength of this study is the inclusion of diverse forms of screen-based activity as well as detailed physical complaints, along with the introduction of a symptom cluster reflecting physical complaints. However, the analyses did not consider differences across electronic devices, online versus offline activities, or time frames (weekdays versus weekends) [91].

Although the study covered nearly 30% of students in the analyzed grades within the studied region, the results cannot be directly generalized to the entire Polish population. Nevertheless, it is reasonable to assume that potential differences from national-level data are relatively small. For example, in the same age group (13–17 years), at least two symptoms out of the eight included in the HBSC-SCL scale were reported by 56.6% of students in the surveyed region of western Poland in 2024, compared with 58.4% in the national HBSC 2022 sample [44].

The use of well-established and validated research instruments for measuring both the severity of complaints and screen time ensures the reliability of the obtained results and facilitates comparisons with other Polish and international studies. In research on complaints reported by school-aged adolescents, physical complaints often receive less attention, with greater emphasis placed on psychological symptoms, sometimes analyzed together with physical symptoms in the context of somatization. Expanding the HBSC-SCL scale with two additional symptoms and developing a new short index of physical complaints associated with screen time represents a novel aspect of this study, which has implications for the practical conclusions and educational recommendations presented below.

At the same time, several limitations should be acknowledged. First, reliance on self-reported data introduces potential reporting and recall bias. In particular, self-reported measures of screen time do not account for the simultaneous use of multiple devices or activities, which may lead to misestimation of actual screen exposure and shape the observed associations.

Second, the cross-sectional design of the study limits the ability to determine the temporal sequence of the observed relationships and precludes causal inference. We have adopted a conventional direction of causality, treating screen time as a determinant of physical health problems. It is to be expected that a number of moderating factors or mediators effects the relationship under investigation and each of these variables has its own determinants. Important factors worth considering in future studies include the presence of chronic conditions, the quality and duration of sleep, and leisure activities that support healthy development.

Third, the study did not include a direct assessment of body posture. Although the observed associations between screen time and musculoskeletal complaints may be partly explained by postural factors, no objective measures of posture were included.

Finally, the relatively modest explanatory power of the models indicates that a substantial proportion of variability in physical complaints remains unexplained. This reflects the multifactorial nature of adolescent health, which is linked to a wide range of interrelated biological, behavioral, and environmental factors [92]. It can be assumed that screen time will not be eliminated from the model explaining the variability of the index under study once other factors are included. It is undoubtedly an important, but not the dominant, predictor of adolescents’ physical well-being.

Despite these limitations, the statistical analyses conducted constitute a strength of the study, as they are diverse and comprehensive in assessing both the significance and strength of the observed relationships. The inclusion of both univariate and multivariate analyses allowed for a more in-depth assessment of the relationships between variables.

### 4.6. Practical Implications and Recommendations

The findings of this study have important practical implications for professionals involved in adolescent health care, including family physicians, pediatricians, psychiatrists, psychologists, orthopedists, ophthalmologists, and physiotherapists. Adolescents’ digital behaviors should be considered when collecting medical histories from patients who report physical symptoms. Health professionals should also be prepared to provide guidance based on established ergonomic principles used in occupational medicine regarding safe computer use, including appropriate body posture, screen distance, and the frequency and duration of breaks [41]. Adolescents should be made aware of the relationship between screen time and overall well-being, encompassing both mental and physical health. This includes understanding the importance of maintaining musculoskeletal health and proper visual function, which is also relevant when using smartphones. Appropriate counseling may help improve health literacy among young people and reduce the risk of physical complaints associated with prolonged use of digital devices.

The findings should also be considered by policymakers and incorporated into health promotion programs. Public debate currently focuses primarily on mental health problems, which have received increasing attention in the context of the adolescent mental health crisis and the growing risk of media addiction. However, physical complaints should not be overlooked, as they may significantly affect young people’s quality of life and well-being both now and in the future [37]. These implications should be interpreted with caution given the observational nature of the study. Such analyses highlight the significance of the problem, but do not provide a framework for preventive measures. Nevertheless, it is important to promote guidelines on the healthy use of smartphones, particularly in the context of risks related to postural disorders, eye problems, headaches and dizziness, as well as sleep disturbances.

This study may also suggest directions for future research on the determinants of psychosomatic complaints. Rather than relying solely on existing instruments, we attempted to adapt measurement tools in response to the determinants examined in this study. When investigating the health effects of adolescents’ excessive use of digital media—a relatively new phenomenon—it is important to monitor additional health outcomes that may not yet be included in existing measurement tools. Future research should also consider emerging patterns of media use and potential health consequences, including those related to the growing use of artificial intelligence. Emphasizing physical complaints adds an important dimension to the ongoing discussion on how to strengthen digital resilience among adolescents [93].

The HBSC-SCL extension will be included in the Polish HBSC 2026 questionnaire. This will make it possible to estimate a more comprehensive model of physical complaints variability, taking into account additional predictors, across a nationwide sample comprising this time four age groups (including 11-year-olds).

## 5. Conclusions

This study demonstrates that screen time is significantly associated with the severity of non-specific physical complaints among adolescents; however, the strength and nature of these relationships vary depending on the type of digital activity. Engagement in social media emerged as the strongest predictor of both individual symptoms and the cumulative burden of complaints, exceeding the association with time spent on computer games, watching video materials, and browsing websites.

The findings confirm that physical complaints during adolescence are multidimensional and often co-occur, with their severity linked to a range of interacting factors. Gender plays an important moderating role in these relationships, with girls reporting a higher burden of complaints and showing a stronger association between screen time and physical symptoms.

These results underscore the need to develop and evaluate preventive interventions that take into account the diversity of digital activities as well as gender- and development-related differences.

## Figures and Tables

**Table 1:** Daily time spent in front of screens by type of activity (%).

Activity	None	0.5–1 h	2–3 h	4–5 h	≥6 h	Mean ± SD
Computer games	13.9	25.3	30.4	17.7	12.7	2.61 ± 2.24
Social media	5.4	27.2	33.3	19.9	14.2	2.89 ± 2.16
TV or videos	9.4	42.7	34.4	9.2	4.3	1.84 ± 1.64
Websites	12.0	69.9	13.3	2.8	2.0	1.04 ± 1.25

**Table 2:** Frequency of physical complaints reported by school-aged adolescents (%).

Symptom	Rarely or Never	About Every Month	About Every Week	More than Once a Week	About Every Day	Mean * ± SD
Headache	37.1	22.5	13.9	14.6	11.9	1.42 ± 1.41
Eye strain	52.6	13.6	9.2	8.9	15.7	1.21 ± 1.53
Backache	37.0	19.9	13.1	11.1	18.9	1.55 ± 1.53
Neck or shoulder pain	47.9	19.4	11.4	9.6	11.7	1.18 ± 1.41
Sleeping difficulties	36.6	16.6	12.5	12.6	21.7	1.66 ± 1.58
Dizziness	53.7	16.7	10.1	8.6	10.9	1.06 ± 1.40

* Mean score on a 0–4 scale.

**Table 3:** Recurrent physical complaints by socio-demographic characteristics.

	More than Once a Week or About Every Day (%)					
	Headache	Eye Strain	Backache	Neck or Shoulder Pain	Sleeping Difficulties	Dizziness
total	26.5	24.6	30.0	21.3	34.3	19.5
Gender						
Boys	16.4	16.6	23.1	16.0	28.0	10.4
Girls	36.6	32.5	36.8	26.6	40.7	28.6
*p*	<0.001	<0.001	<0.001	<0.001	<0.001	<0.001
Cramer’s *V*	0.228	0.185	0.150	0.129	0.134	0.230
Grade						
K7	25.1	22.6	25.3	18.9	33.3	19.9
K9	27.8	25.7	30.7	21.4	34.3	21.7
K11	26.7	25.4	33.9	23.6	35.4	16.9
*p*	0.052	0.007	<0.001	<0.001	0.204	<0.001
Cramer’s *V*	0.025	0.033	0.078	0.047	0.019	0.050
Family affluence						
Low	27.5	25.7	30.6	22.2	34.3	20.1
Average	25.4	23.4	29.4	20.3	33.7	18.5
High	27.6	25.1	30.5	22.5	35.5	21.2
*p*	0.075	0.063	0.474	0.081	0.481	0.038
Cramer’s *V*	0.024	0.025	0.013	0.024	0.013	0.027
Domicile						
Big cities	26.1	25.6	30.5	23.2	36.2	20.6
Small cities	26.2	24.8	30.8	21.9	34.1	19.3
Rural areas	27.2	23.7	28.6	19.4	33.4	19.1
*p*	0.548	0.282	0.114	0.003	0.114	0.361
Cramer’s *V*	0.011	0.017	0.022	0.036	0.022	0.015

**Table 4:** Physical complaints index by respondents’ characteristics.

	Mean	SD	Test *	*p*	Post-Hoc Kruskal–Wallis
Total	6.54	5.15			
Gender					
Boys	4.97	4.60	*Z* = −30.901	<0.001	–
Girls	8.10	5.21			
Grade					
K7	6.14	5.15			K7 & K9: *p* < 0.001
K9	6.65	5.27	*H* = 34.360	<0.001	K7 & K11: *p* < 0.001
K11	6.82	5.01			K9 & K11: *p* = 0.381
Family affluence					
Low	6.69	5.24			Low & Average: *p* = 0.021
Average	6.36	5.05	*H* = 7.664	0.022	Low & High: *p* = 0.698
High	6.71	5.28			Average & High: *p* = 0.029
Domicile					
Big cities	6.72	5.22			Big & Small: *p* = 0.687
Small cities	6.59	5.14	*H* = 9.066	0.011	Big & Rural: *p* = 0.011
Rural areas	6.35	5.13			Small & Rural: *p* = 0.009

* *Z*—Mann–Whitney U test; *H*—Kruskal–Wallis test.

**Table 5:** Association between individual physical complaints and screen time estimated using multivariate general linear models *.

Dependent Variable		Computer Games	Social Media	TV or Videos	Websites	*R*^2^ **
Headache	*β*	0.022	0.084	0.006	0.054	
	*p*	0.002	<0.001	0.541	<0.001	0.110
	partial *η*^2^	0.001	0.015	<0.001	0.002	(0.053)
Eye strain	*β*	0.034	0.061	0.035	0.043	
	*p*	<0.001	<0.001	0.001	0.002	0.068
	partial *η*^2^	0.002	0.006	0.001	0.001	(0.032)
Backache	*β*	0.031	0.104	0.050	0.024	
	*p*	<0.001	<0.001	<0.001	0.074	0.079
	partial *η*^2^	0.002	0.019	0.003	<0.001	(0.051)
Neck or shoulder pain	*β*	0.030	0.056	0.035	0.080	
	*p*	<0.001	<0.001	<0.001	<0.001	0.051
	partial *η*^2^	0.002	0.006	0.001	0.004	(0.032)
Sleeping difficulties	*β*	0.043	0.103	0.022	0.048	
	*p*	<0.001	<0.001	0.043	<0.001	0.063
	partial *η*^2^	0.003	0.017	<0.001	0.001	(0.045)
Dizziness	*β*	0.026	0.098	0.007	0.071	
	*p*	<0.001	<0.001	0.452	<0.001	0.118
	partial *η*^2^	0.002	0.020	<0.001	0.004	(0.063)

* Adjusted for gender, grade, place of residence, and family affluence (FAS) as factors; ** *R*^2^—coefficient of determination for the adjusted model (and the unadjusted model in parentheses).

**Table 6:** Model of determinants of variability in the 5-item summary index of physical complaints *.

Independent Variables	*Β*	SE	95% CI Lower	95% CI Upper	*p*	Partial *η*^2^
Constant	6.13	0.20	5.74	6.53	<0.001	0.094
Gender						
Boys	−2.88	0.11	−3.10	−2.67	<0.001	0.070
Girls (ref.)						
Grade						
K7	−0.57	0.14	−0.84	−0.31	<0.001	0.002
K9	−0.22	0.14	−0.49	0.05	0.114	<0.001
K11 (ref.)						
Domicile						
Big city	0.25	0.14	−0.02	0.52	0.068	<0.001
Small city	0.23	0.12	0.01	0.46	0.042	<0.001
Rural areas (ref.)						
Family affluence						
Low	−0.03	0.15	−0.33	0.27	0.857	<0.001
Average	−0.25	0.14	−0.53	0.04	0.087	<0.001
High (ref.)						
Screen time (continuous)						
Computer games	0.15	0.02	0.11	0.20	<0.001	0.004
Social media	0.40	0.03	0.35	0.45	<0.001	0.026
Videos	0.11	0.03	0.04	0.17	0.002	0.001
Websites	0.30	0.04	0.21	0.38	<0.001	0.005

* *R*^2^ = 0.153; ref. = reference group.

**Table 7:** Association between the variability of the five-item summary index of physical complaints in gender-specific models *.

	Boys			Girls		
Independent Variables	*Β*	SE	*p*	*β*	SE	*p*
Constant	3.55	0.27	<0.001	5.91	0.27	<0.001
Grade						
K7	−0.73	0.19	<0.001	−0.47	0.19	0.015
K9	−0.54	0.19	0.005	0.03	0.19	0.878
K11 (ref.)						
Domicile						
Big city	0.24	0.19	0.199	0.26	0.20	0.197
Small city	0.11	0.16	0.466	0.33	0.17	0.044
Rural areas (ref.)						
Family affluence						
Low	0.17	0.21	0.422	−0.18	0.22	0.409
Average	−0.15	0.20	0.444	−0.32	0.20	0.122
High (ref.)						
Screen time (continuous)						
Computer games	0.13	0.04	<0.001	0.17	0.03	<0.001
Social media	0.37	0.04	<0.001	0.42	0.04	<0.001
TV and videos	0.06	0.04	0.217	0.16	0.05	0.001
Websites	0.38	0.06	<0.001	0.22	0.06	0.001

* *R*^2^—boys: 0.068; girls: 0.062; ref. = reference group.

## Data Availability

The data presented in this study are not publicly available due to privacy and ethical restrictions.

## Appendix A

**Table A1:** Mean daily screen time (hours) by type of activity and socio-demographic characteristics.

	Computer Games	Social Media	TV or Videos	Websites
Total	2.61 ± 2.24	2.89 ± 2.16	1.84 ± 1.64	1.04 ± 1.25
Gender				
Boys	3.19 ± 2.13	2.24 ± 2.00	1.98 ± 1.72	1.02 ± 1.28
Girls	2.17 ± 2.23	3.50 ± 2.16	1.71 ± 1.57	1.01 ± 1.22
Mann–Whitney *Z*	−25.992	−29.690	−7.920	−0.247
*p*	<0.001	<0.001	<0.001	0.805
Grade				
K7	2.79 ± 2.18	2.68 ± 2.16	1.87 ± 1.66	0.96 ± 1.25
K9	2.75 ± 2.31	3.12 ± 2.21	1.79 ± 1.65	1.02 ± 1.26
K11	2.14 ± 2.15	3.06 ± 2.11	1.83 ± 1.60	1.12 ± 1.22
Kruskal–Wallis *H*	169.814	100.033	5.687	103.904
*p*	<0.001	<0.001	0.058	<0.001
Family affluence				
Low	2.77 ± 2.31	2.92 ± 2.23	1.96 ± 1.74	1.04 ± 1.26
Average	2.56 ± 2.19	2.91 ± 2.16	1.79 ± 1.57	1.00 ± 1.18
High	2.54 ± 2.24	3.02 ± 2.12	1.71 ± 1.65	1.03 ± 1.35
Kruskal–Wallis *H*	14.166	5.764	26.120	2.590
*p*	<0.001	0.056	<0.001	0.274
Place of residence				
Large cities	2.74 ± 2.28	2.91 ± 2.24	1.89 ± 1.70	1.00 ± 1.27
Small towns	2.63 ± 2.24	2.92 ± 2.15	1.82 ± 1.62	1.06 ± 1.27
Rural areas	2.57 ± 2.22	2.94 ± 2.18	1.81 ± 1.64	0.98 ± 1.20
Kruskal–Wallis *H*	6.349	1.088	3.005	12.949
*p*	0.042	0.580	0.223	0.002

**Table A2:** Psychometric evaluation of alternative physical complaints scales using confirmatory factor analysis (CFA).

	Index 1	Index 2	Index 3	Index 3a *	Index 4
Symptoms included	Headache; Eye strain; Backache; Neck/shoulder pain; Sleeping difficulties; Dizziness	Headache; Eye strain; Backache; Sleeping difficulties; Dizziness	Headache; Eye strain; Neck or shoulder pain; Sleeping difficulties; Dizziness	Same as Index 3	Headache; Eye strain; Neck or shoulder pain; Dizziness
CMIN	218.718	15.717	9.973	6.680	14.798
NFI	0.881	0.992	0.995	0.997	0.996
RFI	0.777	0.984	0.989	0.993	0.987
TLI	0.778	0.985	0.991	0.994	0.988
CFI	0.881	0.992	0.995	0.998	0.996
SRMR	0.0568	0.0149	0.0120	0.0085	0.0123
RMSEA	0.154	0.040	0.031	0.025	0.039
90% CI	0.148–0.160	0.032–0.048	0.024–0.039	0.016–0.034	0.027–0.052
Cronbach’s α	0.786	0.749	0.744	0.744	0.712

* Correlated error terms for headache and sleeping difficulties.
